# Benefit of educational feedback for the use of positive expiratory pressure device

**DOI:** 10.1590/bjpt-rbf.2014.0111

**Published:** 2015-09-01

**Authors:** Gregory Reychler, Manon Jacquemart, William Poncin, Anne-Sophie Aubriot, Giuseppe Liistro

**Affiliations:** 1 Institut de Recherche Expérimentale et Clinique (IREC), Pôle de Pneumologie, ORL & Dermatologie, Université Catholique de Louvain (UCL), Brussels, Belgium; 2 Service de Pneumologie, Cliniques Universitaires Saint-Luc, Brussels, Belgium; 3 Service de Médecine Physique et Réadaptation, Cliniques Universitaires Saint-Luc, Brussels, Belgium; 4 Centre de Référence pour la Mucoviscidose, Cliniques Universitaires Saint-Luc, Brussels, Belgium

**Keywords:** physical therapy, chest, education, device

## Abstract

**BACKGROUND:**

Positive expiratory pressure (PEP) is regularly used as a self-administered airway clearance technique.

**OBJECTIVE:**

The aim of this study was to evaluate the need to teach the correct use of the PEP device and to measure the progress of the success rate of the maneuver after training.

**METHOD:**

A PEP system (PariPEP-S Sytem) was used to generate PEP in 30 healthy volunteers. They were instructed by a qualified physical therapist to breathe correctly through the PEP device. Then they were evaluated during a set of ten expirations. Two other evaluations were performed at day 2 and day 8 (before and after feedback). The mean PEP and the success rate were calculated for each set of expirations. The number of maneuvers needed to obtain a correct use was calculated on the first session.

**RESULTS:**

An optimal PEP was reached after 7.5 SD 2.7 attempts by all subjects. Success rates and mean pressures were similar between the different sets of expirations (p=0.720 and p=0.326, respectively). Pressure variability was around 10%. After one week, 30% of subjects generated more than two non-optimal pressures in the set of ten expirations. No difference in success rate was observed depending on the evaluations.

**CONCLUSION:**

This study demonstrates that good initial training on the use of the PEP device and regular follow-up are required for the subject to reach optimal expiratory pressure.

## Introduction

Airway clearance techniques are frequently recommended in respiratory disease. Adherence, which is defined by the degree of concordance (or agreement) between the health professional's recommended therapy and the patient's actual behaviour^1^, can represent a problem for patients even if airway clearance techniques seem to have a satisfactory rate of adherence^2^^,^ 3. Two key factors contributing to the lack of adherence regarding physical therapy are the need of daily assistance from a skilled therapist^4^ and the time-consuming aspect of this part of the treatment due to the duration of the sessions and their high frequency^5^.

For these reasons, several self-administered airway clearance techniques have been developed with similar efficacy to manual ones^6^^-^ 9 and a tendency to higher individual preference^10^. Among these are a range of positive expiratory pressure (PEP) devices, which were perceived as more convenient than manual techniques^11^. They provide constant back-pressure into the airways during the expiration. The increased pressure is transmitted to the airways and acts to prevent airway closure and reduce gas trapping in the lung^12^. Obstructed airways are reopened by increased collateral ventilation^13^. In addition to holding the airways open and a higher generated expiratory airflow in the small airway, PEP aims to promote mucus mobilization based on transmitted flow into the airways^14^. Moreover, PEP appeared to counteract the effect of gravity on spatial ventilation distribution^15^. In the original description of the PEP mask, the patients were breathing against a resistance generating a positive expiratory pressure of 10 to 20 cmH_2_O^16^.

In daily routine, the PEP device is frequently proposed to patients suffering from lung diseases^17^^-^ 19 as a self-administered airway clearance technique, but often neither instruction nor regular follow-up are provided. It is the most frequently used airway clearance device in cystic fibrosis (CF)^20^. It provides the benefits of improved airway clearance in the patient's home, potentially reducing demands on healthcare resources^21^ and cost^9^. Even if the results in the literature are heterogenous^20^^,^ 22 ^,^ 23, long-term studies in CF patients have shown that it provides more benefit than postural drainage in conjunction with percussions and vibrations^24^ or Flutter^25^. Most importantly, the use of PEP devices is less time-consuming and sometimes more comfortable for the patient than other techniques, which explains why PEP is regularly preferred over other techniques^10^^,^ 20.

As demonstrated for manual airway clearance techniques^26^, performance variability could be related to mechanical airway clearance techniques. Indeed, Flutter device inclination and expiratory flow of the patient modify its operational physical variable such as mean pressure and flow, oscillation frequency, and flow amplitude. This variability influences the effects on airway clearance^27^. Moreover, the peak expiratory flow rate and tidal expiratory volume variation were 34% and 29%, respectively, when using a PEP mask in 18 cystic fibrosis patients after standardized instruction given by the same physical therapist^28^. Suboptimal training was also noted during IMT training for 30% of the patients included in a program^29^. Therefore, even if the need to check the patient's technique seems evident with PEP devices, its effect on the maneuver was never investigated.

Assuming that the correct use of a technique is a key component in efficacy, the aim of this study was to evaluate the need to teach the optimal use of PEP device and to measure the evolution in the time of the success rate of the maneuver after education.

## Method

### Subjects

Thirty healthy non-smoking volunteers were recruited for the study in our university staff. They were unfamiliar with the device and its use. They signed a written informed consent form in accordance with the Declaration of Helsinki and the current guidelines for Clinical Good Practice. The study was previously approved by the Institutional Medical Ethics Committee (Comission d'éthique biomédicale Hospitalo-Facultaire, UCL, Brussels, Belgium) (B4032011110043) and was registered as a clinical trial (NCT02031926). Exclusion criteria included a history or evidences of pulmonary disease, Attention-Deficit/Hyperactivity (ADHD) or cognitive disorder, contraindications to the use of PEP and previous use of the device.

### Procedure

A PEP system (PariPEP-S System, PARI GmbH, Germany) was used to generate a positive expiratory pressure. The subjects were seated on a chair and their fingers were placed around the mouthpiece. They wore a nose clip as recommended by the manufacturer. The pressure was controlled by a manometer connected to the expiratory valve of the PEP system. All of the subjects were instructed by a qualified physical therapist to breathe through the mouthpiece, take a deep breath, and exhale slightly and actively at a comfortable flow rate. A physical demonstration was performed by the physical therapist. When expiring through the PEP system, the generated pressure was measured in mbar and converted into cmH_2_O. The chosen resistor for each participant was the resistor generating an expiratory pressure between 10 and 20 cmH_2_O. The inner diameter of the resistors ranged from 2.0 mm to 4 mm after adaptation.

### Outcomes

The pressure generated during expirations was measured with a manometer (PARI, PARI GmbH, Germany). It records pressures ranging from 0 to 100 mbar. The mean pressure was calculated for each set of ten expirations. The number of maneuvers needed to obtain a correct use was calculated at the first session. The success rate was calculated by the number of expirations generating a pressure higher than 10 cmH_2_0 and lower than 20 cmH_2_O. It was expressed in percentage of ten expirations for each set. The lowest and the highest generated pressures were noted in each set of ten expirations.

### Design

The study included three sessions. During the first session (S1), the optimal resistor for correct use of the PE device was determined for each subject. The subjects performed expirations through the device, and after each maneuver, they received feedback from the physical therapist regarding the generated pressure. The number of attempts needed to reach success was noted. Then the subjects were asked to perform ten maneuvers without feedback (E1). The next day, a second session (S2) was completed. At the beginning and without instruction, the subject performed ten expirations (E2) through the same resistor as the day before. After this set of expirations, feedback was provided by the physical therapist to correct the technique, if necessary. A second set of ten repetitions (E3) was performed. One week later, a third session (S3) equal to the second one (E4 and E5) was performed to verify if correct use of the device was maintained with time.

### Statistical methods

The sample size needed to detect a 25% difference in success rate at a 5% significance level for a two-sided comparison with 80% power was determined (n=27). Statistical tests were performed using SPSS Statistics 22.0 (IBM Company). Results are expressed as median and interquartile range or mean, standard deviation (SD) and coefficients of variation depending on the normality of the distribution. Confidence interval was used as indicator of the precision of the estimates. Normality of the distributions was verified by the Kolmogorov-Smirnov test. Friedman's test was used to detect differences in pressure and success rate across multiple attempts. The Wilcoxon signed-rank test was used to compare differences between the success rates of the sessions before and after teaching. A p value less than 0.05 was considered as statistically significant.

## Results

The anthropometric parameters of the subjects are shown in Table 1. All of the patients completed the study and succeeded in generating optimal pressure (between 10 and 20 cmH_2_O) on the initial visit. The number of attempts to obtain this pressure on the first session was 7.5 SD 2.7.

**Table 1.** Anthropometric data of the 30 subjects.

Age (yr.)	22 (2.65)
Sex (F/M)	15/15
Weight (kg)	66.2 (12.9)
Height (cm)	170.3 (10.3)
IMC (kg/m2)	22.7 (3.0)

Shown as mean (SD).

The success rates and the mean pressures of the different sets of expirations through the device are shown in Table 2. They were similar between the different sets of expirations (p=0.720 and p=0.326, respectively). With 30 patients, the study has a 97% power to detect a change of at least one standard deviation between two sets.

**Table 2.** Success rate and pressure obtained by the 30 subjects during the different sets.

Set	Success rate (%)	Pressure (cmH_2_O)			
	Median (IQR)	Mean (SD)	Coefficient of variation	Min.	Max.
E1	100 (90-100)	14.7 (2.4) [13.8;15.6]	0.09	9	27
E2	100 (90-100)	15.0 (3.4) [13.7;16.3]	0.11	5	40
E3	100 (90-100)	14.7 (2.9) [13.6;15.8]	0.10	9	30
E4	100 (80-100)	14.1 (2.9) [13.1;15.2]	0.11	5	35
E5	100 (87.5-100)	14.3 (2.9) [13.2;15.4]	0.09	8	24
Coefficient of variation		0.025			

Maneuver without feedback (E1). Set of ten expirations without feedback or instruction (E2). Set of expirations after correction of the technique on day 1 (E3). Set of ten expirations without feedback and instruction on day 8 (E4). Set of ten expirations after correction of the technique on day 8 (E5). IQR: interquartile range 25%-75%. Confidence interval is expressed in brackets.

Similarly, the lowest and the highest pressures in a set of expirations were similar between sets (p=0.865 and p=0.738, respectively). The variability of the generated pressures was around 10%.

The minimal and maximal pressures generated by the subjects were out of range for all of the sets. Nine subjects (30%) generated at least one non-optimal expiratory pressure during the set of expirations at the initial session of the second visit (E2). Two subjects (7%) used the device inadequately in all of the expirations of this set (E2). After one week (E4), nine subjects performed more than two non-optimal pressures in the set of ten expirations.

No difference in success rate was observed between E2 - E3 (p=0.185; Table 2) and E4 - E5 (p=0.217).

## Discussion

This study showed that all of the subjects generated the recommended range of pressure with the PEP mask; however, training is necessary to obtain this pressure. Moreover, the success rate remained similar after one week without supervision even if non-optimal pressures were found in a set of expirations.

All of the subjects reached a mean optimal expiratory pressure after an initial training session, and the variability of the generated pressure was low. This means that the technique is feasible in these conditions. The number of repetitions needed to ensure optimal pressure^16^^,^ 25 ^,^ 30 was high in spite of constant feedback, which implies that initial supervision is necessary even if the aim is the autonomy of the patient. With this supervision, however, a maximal success rate was acquired.

Although the success rate and the variability of the generated pressures were constant for all sets, the minimal and maximal pressures generated were systematically inadequate. This indicates that at least one maneuver was outside the range of recommended pressures, regardless of the set. Moreover, the check-up of proper device use on the second day was necessary for 30% of the subjects. On this second visit, two subjects out of 30 performed all the ten expirations inadequately. Therefore, regular follow-up is necessary even if the overall success rate remained similar.

Surprisingly, adherence to the correct use of PEP devices has never been observed before even though the need to check the maneuver has been previously suggested^25^. However, our results are not so surprising. Indeed, even if inhalers are completely different from PEP devices regarding the way of functioning, the need and the importance of regular instructions has also been previously observed for these devices^31^. The misuse of inhalers varies between 4 and 94% of patients^32^. Despite the great differences in way of functioning, we hypothesized similar results regarding the success of use of PEP devices, and this was verified in our study.

Regarding training, simply reading written instructions did not appear to be sufficient. The physical demonstration is a central component for educating patients^33^. One study showed that regular training, including practical demonstration provided by the healthcare staff, is the most efficient strategy for reducing errors in inhaler use compared to a leaflet or an oral explanation^34^ even if these elements are also necessary. The basics of effective education and, consequently, of effective treatment are simplification, demonstration, and repetition of the maneuver^35^. This principle must be transposed to the PEP device. This model was applied in our study, and our results confirm that it is possible to obtain high adherence in each session with a low variability in time (less than 5%). Even if there was no difference in success rate between the sessions before and after training on the second and third visits, we observed a tendency for improvement.

Among many other factors, the lack of adequate patient training about device use^36^ and the low level of the caregiver's skills^37^ have been previously related to poor adherence to inhalation. We hypothesize that the mechanism for the PEP device or other chest physical therapy techniques is similar. Moreover, Wong showed a significant relationship between physical therapists' characteristics or clinical experience and technique performance^38^. It seems to justify the need for an experienced physical therapist to teach the technique. For this reason, in our study, the physical therapist involved in the training of the subjects was qualified and familiar with PEP use. Regarding our results, it would be interesting to observe the knowledge of the physical therapist population regarding the guidelines for PEP device use.

Some methodological aspects of the study should be addressed. Only young healthy subjects were recruited in this non-probabilistic sample. However, it seems improbable that health status influences the adherence to the device comparatively to healthy subjects. Indeed, even if studies have suggested in the past that adherence to chest physical therapy might be low^5^, a good adherence regarding physical therapy was recently demonstrated in cystic fibrosis^3^. Moreover, the use of positive expiratory pressure devices has a high level of agreement in cystic fibrosis patients aged 16 years and older between physical-therapist recommended ACT technique and self-reported subject adherence^2^. It could also be argued that the subjects' age is a potential influence and it needs to be verified in another study. The short follow-up is another limitation of the study and it would be interesting to investigate a longer period. However, as we showed the necessity of regular follow-up in the short term, the need could be potentially higher in the long term. Finally, obstructive lung diseases are associated with flow reduction, and it could be difficult to reach the optimal pressure with the PEP device. However, it was demonstrated that these patients can generate pressures higher than 40 cmH_2_O^39^.

In conclusion, this study demonstrates that good initial training on the use of the PEP device and regular follow-up are required for the subject to reach recommended expiratory pressure.
